# Advances in recombinant antibody manufacturing

**DOI:** 10.1007/s00253-016-7388-9

**Published:** 2016-03-03

**Authors:** Renate Kunert, David Reinhart

**Affiliations:** Vienna Institute of BioTechnology, Department of Biotechnology, University of Natural Resources and Life Sciences, Vienna, Muthgasse 11, 1190 Vienna, Austria

**Keywords:** Chinese hamster ovary (CHO), Human embryonic kidney (HEK), NS0, PER.C6, Monoclonal antibody (mAb) manufacturing, Mammalian expression systems, Process advances, Optimization strategies

## Abstract

Since the first use of Chinese hamster ovary (CHO) cells for recombinant protein expression, production processes have steadily improved through numerous advances. In this review, we have highlighted several key milestones that have contributed to the success of CHO cells from the beginning of their use for monoclonal antibody (mAb) expression until today. The main factors influencing the yield of a production process are the time to accumulate a desired amount of biomass, the process duration, and the specific productivity. By comparing maximum cell densities and specific growth rates of various expression systems, we have emphasized the limiting parameters of different cellular systems and comprehensively described scientific approaches and techniques to improve host cell lines. Besides the quantitative evaluation of current systems, the quality-determining properties of a host cell line, namely post-translational modifications, were analyzed and compared to naturally occurring polyclonal immunoglobulin fractions from human plasma. In summary, numerous different expression systems for mAbs are available and also under scientific investigation. However, CHO cells are the most frequently investigated cell lines and remain the workhorse for mAb production until today.

## Historical and scientific background

Monoclonal antibodies are the largest group of recombinant proteins used not only for human therapy but also for in vivo imaging of different types of diseases.

In the mid-1970s, the foundation for the commercial success of antibodies was laid by Köhler and Milstein who developed the method for immortalization of B lymphocytes for the purpose of monoclonal antibody production (Köhler and Milstein 1975). Isolated primary lymphocytes, differentiated and matured in the body, are fused with immortal lymphoblastoid cells, the so-called myeloma cells. Myeloma cells had already been cultivated since the 1960s, and auxotrophic cell strains were established after intensive treatment with chemicals or radiation. These early cell lines were then used for scientific evaluation and were also needed for the newly developed hybridoma technology enabling the discrimination between fused and unfused cells. Despite similar attempts at expressing other products with immortalized, differentiated cells being undertaken, the upcoming pharma industry in particular soon recognized the potential of mAbs as future blockbusters. The reason was that hybridoma technology was published in Nature in 1975 and therefore free of IP rights (Köhler and Milstein 1975). Most importantly, individual antibodies are considered to be individual products and can be patented to generate market exclusivity. Consequently, the first protein from animal cell culture for human application was OKT3, a mouse mAb directed against T lymphocytes to prevent host versus graft reaction in kidney transplantation (Smith 1996).

The next generation of mAb-producing cell lines was driven by the problems of hybridoma instability and the development of human anti-mouse antibodies (HAMA) by the patients. Progress in molecular biology techniques and the feasibility of directed genetic manipulation of mammalian cells enabled the production of mAbs by recombinant cell lines instead of less-defined hybridoma cells. The 1990s were dominated by the development of chimeric antibodies with human constant immunoglobulin regions and mouse variable regions to prohibit HAMA responses in patients. To delete the remaining T cell epitopes located on the variable regions of mAbs, humanization strategies were developed by grafting the antigen-binding domains (located on the complementary determining regions and determined by three heavy chain and three light chain peptide loops) to human framework regions of existing antibodies or to the nearest related germline regions. An alternative humanization strategy called resurfacing is based on the identification of surface accessible residues to evaluate their necessity for antigen binding or their potential for generating a T cell epitope (Pedersen et al. 1994; Roguska et al. 1994). However, the best method to generate a human applicable mAb is still to start from original human genes. Those might be obtained from antigen-primed donors as well as naïve or immune libraries which are commercially available. In this case, IP rights need to be charged for the technological package.

The dominating production system for mAbs is a recombinant expression in cellular systems. Not only the costs of the final drug product is the most important decision-driving parameter for a production system but also downstream procedures have to be evaluated carefully since they contribute significantly to meeting all regulatory demands and clinical efficacy. Product expense is a consequence of cost of goods and available capacity of the production facility, which might differ for the individual cell systems. A major impact is raised by downstream procedures and product quality assurance steps which cause significant costs even when cheap technologies with prokaryotic systems and lower eukaryotes are applied. In this review, the CHO production system is compared to alternative systems by means of production capacity and product quality.

Production systems under development, often already for decades, are transgenic plants and animals as well as in vitro translation systems. The difficulties which are encountered in those systems are most often insufficient product titers (transgenic animals), unwanted glycosylation patterns or proteolytic activity (both predominantly envisaged in transgenic plants), rendering the downstream procedures complicated and inefficient. Besides the availability of these expression systems, antibodies for human application are isolated by human plasma fractionation as initially developed during World War II. The industrial scale of single facilities is in the range of several thousand liters run in shift operations (Robert and Hotchko 2013). Frozen or fresh plasma pools are fractionated to isolate valuable components of the human serum proteome. One of them is the immunoglobulin fraction which is sold as plasma-derived intravenous immunoglobulin (IVIG), which is a polyvalent product generated from 10,000 to 40,000 donors. Plasma donations containing elevated levels of clinically important antibodies are preferably used for the production of ‘hyperimmune’ or ‘specific’ IgG products, such as anti-hepatitis B, anti-tetanus, anti-cytomegalovirus, or anti-rabies. To get an estimate of the production capacity, nearly 100 tons of IVIG are used in clinical applications per year. This means that the human individual also represents a factory for commercial antibody production, and huge amounts are isolated and purified from a worldwide cohort of millions of donors.

### The factor “production efficacy-yield” in mAb production

Production yield is always a matter of numerous factors of the complex procedure of mAb manufacturing and therefore needs to be split into different unit operations to evaluate the critical steps. One major step is the upstream process, starting from the frozen vial of the producer clone and ending with the harvest of the culture broth. Critical parameters in this procedure are (i) the time until a desired cell density is reached, determined by the specific growth rate (μ) of the cells; (ii) the duration of the production phase enabling an accumulation of recombinant protein from a high-density and viable culture; and (iii) the obtainable product titer determined by the specific production rate (qP) and the overall process duration.

### Host cell lines for mAb production and host cell engineering

The most prominent host cell lines for recombinant mAb expression are CHO, NS0, Sp2/0, HEK293, and PER.C6. However, most literature is available for CHO cells, which are still the workhorse for 70 % of today’s industrially produced protein therapeutics (Jayapal et al. 2007). In this context, it should be noted that mAbs approved for human therapy are only produced in CHO, NS0, and Sp2/0 (Dumont et al. 2015). Reviewed literature investigating high-producing cell lines is often not available, sometimes for IPR reasons or because only the process or the medium is of major interest and the cell line serves only as a tool. Table 1 gives an overview of scientific publications without considering therapeutic application.

**Table 1:** Relevant bioprocess parameters for different mAb expression systems

Cell line	Peak cell concentration	Specific growth rate	Specific productivity	Yield	STY	Process type	Reference
	[10^6^ cells/mL]	[per day]	[pg/cell/day]	[g/L]	[mg/(L × day)]		
CHO	8.5	0.7	>50	0.9	121	Batch	Reinhart et al. (2015)
	23.9	0.7	>50	>5.8	345	Fed-batch	Reinhart et al. (2015)
	1.3	0.8	>2	0.04	2	Batch	Lee et al. (2005)
	27–33.5	n.a.	>2	0.09–0.015	58–72	Perfusion	Lee et al. (2005)
	>10	0.7	57	>1	85	Fed-batch	Zboray et al. (2015)
	25; 26	n.a.	35; 49	10; 13	530; 730	Fed-batch	Huang et al. (2010)
	n.a.	n.a.	n.a.	9.8	470	Fed-batch	Luan et al. (2006)
	>200	n.a.	10–15	n.a.	n.a.	Perfusion	Clincke et al. (2011)
	<110	n.a.	9–13	n.a.	n.a.	Perfusion	Clincke et al. (2013)
PER.C6	5	0.5	n.a.	0.3–0.5	42	Batch	Jones et al. (2003)
	>4	0.5	14–24	0.39	n.a.	Batch	De Kruif et al. (2010)
	n.a.	n.a.	n.a.	8	n.a.	Fed-batch	Kuczewski et al. (2011)
	≥150	n.a.	n.a.	27^a^	n.a.	Perfusion^a^	Kuczewski et al. (2011)
HEK 293^b^	n.a.	n.a.	n.a.	≤0.3	43	Batch	Hasegawa et al. (2014)
	>2	n.a.	20	0.2	20	Batch	Nallet et al. (2012)
	2–4	n.a.	5–10	0.12–0.14^c^	24–28	Batch	Jäger et al. (2013)
	6–8	n.a.	5–10	0.1–0.6^c^	<100	Fed-batch	Jäger et al. (2013)
NS0	0.6–1.0	0.3–0.6	20–50	0.1–0.2	13–17	Batch	Ho et al. (2012)
	2.3	0.6–1.0	8–20	0.07	14	Batch	Spens and Häggström (2007)
	2.3–9.9	1.0	8–20	0.1–0.8	19–67	Fed-batch	Spens and Häggström (2007)
	2.4	0.5	1.9–13.4	0.24	17	Fed-batch	Stansfield et al. (2007)

Values for the specific growth rate refer to the exponential growth phase

*n* *.a.* not available

^a^Product and cell retention in an extreme density bioreactor

^b^Transient mAb expression

^c^Product was a scFv-Fc antibody fragment

CHO cells were used as an expression system for the first approved recombinant biopharmaceutical (tissue plasminogen activator; t-PA) in 1986. Since then, CHO cells have clearly remained the preferred choice for recombinant protein expression. Despite other mammalian expression systems being available, CHO cells have clearly been at the center of attention for research on optimizing recombinant protein expression. Therefore, CHO-based mAb production processes have matured considerably and typically reach the highest product titers with ∼1 g/L in batch and 1–10 g/L in fed-batch processes (Table 1).

The murine cell lines NS0 and Sp2/0 are also used for recombinant mAb production despite expressing the two predominant immunogenic glycan epitopes for humans (Jefferis 2009), galactose-alpha1,3-Gal (alpha-gal), and N-glycolyl-neuraminic acid (Neu5Gc). Both epitopes are absent in CHO and baby hamster kidney (BHK) cells (both of hamster origin). In reported batch cultures, NS0 cells typically reached up to tenfold lower cell and mAb concentrations than CHO-based batch processes (Table 1). Despite achieving relatively high specific productivities of 20–50 pg/cell/day, published mAb titers range from 0.1 to 0.2 g/L in batch cultures and from 0.1 to 0.8 g/L in fed-batches.

Human embryonic kidney (HEK) and PER.C6 cells are often discussed for human identical glycosylation. However, especially for the production of mAbs, the in vivo heterogeneity has to be considered since in distinct cases mAbs from CHO cells showed higher sialylation compared to HEK cells (Croset et al. 2012). HEK293 cells are especially applied for transient gene expression to harvest a protein within a few days after DNA delivery. At present, transiently transfected cells reach mAb expression levels only in the low tens to hundreds of milligrams per liter range during batch cultivation. In fed-batches, mAb titers up to 0.6 g/L were reported for a scFv-Fc antibody fragment (Jäger et al. 2013). Regarding PER.C6 cells, reported product yields are more in the range of CHO-based processes with mAb titers of 0.5 g/L (batch), 8 g/L (fed-batch), and even 27 g/L (perfusion) as shown in Table 1. Although this is impressive, such high mAb concentrations at harvest were achieved due to product retention and concentration during perfusion in the bioreactor (Kuczewski et al. 2011).

The process improvements, which are often only empirically described, are escorted by achievements in vector design and genetic engineering of host cell lines. Vectors with incorporated scaffold/matrix attachment regions (S/MAR) (Zahn-Zabal et al. 2001; Kim et al. 2004; Girod et al. 2005) or ubiquitous chromatin opening elements (UCOEs) (Benton et al. 2002) are designed to provide an “active” configuration of chromatin loops surrounding the transferred gene of interest. Alternatively, bacterial artificial chromosomes (BACs) are DNA transfer vectors carrying a whole eukaryotic locus with all the elements controlling the expression of a gene. Thus, the transgene on the BAC is surrounded by an open/permissive chromatin loci and is not affected by the chromatin environment of the integration site (Kunert and Casanova 2013).

Host cell engineering aims to increase the duration of a (fed-) batch process in the bioreactor. Apoptosis, the major event of different cell death possibilities, is addressed in numerous studies to develop apoptosis-resistant cell lines by different strategies such as anti-apoptotic Bcl-2-like proteins (García-Sáez 2012), the suppression of pro-apoptotic factors, or the inhibition of the p53 tumor suppressor protein (Arden et al. 2007). Similarly, human telomerase reverse transcriptase (hTert) was overexpressed in recombinant CHO cells to induce the proliferation and promote apoptosis resistance (Crea et al. 2006). While the above-mentioned points aim to improve the growth and transcription potential of the cell, other authors aimed to improve the translational capacity of the cell by overexpression of chaperones for efficient maturation of the recombinant and often complex proteins or to reduce the unfolded protein response (UPR) to eliminate apoptosis events as a result of cellular stress (Davis et al. 2000; Chung et al. 2004; Borth et al. 2005; Tigges and Fussenegger 2006; Mohan and Lee 2010). One additional limit of heterologous protein expression might be the secretion pathway, starting from the translocation of the nascent polypeptide chain into the endoplasmatic reticulum (ER). Improper signal peptide cleavage from the antibody chain can lead to light chain aggregation. To reduce this, Le Fourn et al. (2014) overexpressed SRP14, a protein of the signal recognition particle (SRP), and thereby improved the specific productivity of difficult-to-produce antibodies several-fold.

In addition to complete mAbs, also corresponding fragments like scFv, scFvFc, Fab, and F(ab)_2_ are also used in clinical applications and will be paid increasing attention when the diversity of alternative diagnostic and therapeutic drugs is boosted. Such mAb variants are often produced in lower eukaryotes to circumvent technological difficulties and the risk of contamination since high-cell densities are quickly reached due to faster cell propagation. Among the different yeast systems, *Pichia pastoris* is often preferred to *Saccharomyces cerevisiae* for its ability to grow on different carbon sources (including methanol), chemically defined media and the product secretion to the extracellular environment (Macauley-Patrick et al. 2005). The ability of yeast cells to perform post-translational modifications significantly reduces the burden on downstream procedures compared to bacterial systems. The resistance of *P. pastoris* to methanol and the decreased fermentative metabolic pathway enable their cultivation to cell densities above 100 g/L dry cell mass in reusable stainless steel systems (Cregg 2007; Gurramkonda et al. 2009). Such high cell density cultures (HCDC) are often also cost-intensive, and increased concentrations of extracellular proteases might result from increased cell death (Cregg et al. 2000; Shi et al. 2003; Daly and Hearn 2005). Due to the envisaged limits in HCDC, the factor of improving specific protein production rates is the main focus of improving recombinant protein production in yeast cells. Besides the commonly applied codon optimization strategies, coexpression of helper factors like HAC (bZIP transcription factor HAC-1 is involved in the UPR) and SEC4 (involved in vesicular transport) or chaperones actively supporting protein maturation in the ER have been suggested and applied with different degrees of success (Gasser et al. 2006; Damasceno et al. 2007). Therefore, the specific growth rate, maximum cell densities as well as process time shall be compared in a rough approximation in the following section. Here, we want to compare the CHO system with the methylotrophic yeast *P. pastoris* since both systems secrete the recombinant product into the culture supernatant. Both expression systems are predominantly grown in synthetic chemically defined media. Also, downstream procedures are comparable even though increasingly high cell densities may considerably challenge the cell separation step in the *Pichia* system. For realistic comparison of the two systems, the most important parameters describing the exponential growth phase up to the stationary phase of a batch culture are summarized in Table 2.

**Table 2:** Growth parameters and maximum cell densities of a batch process of *P. pastoris* and CHO cells.

Parameter	*Pichia pastoris*	CHO cells
Calculated cell mass^a^ [ng/cell]	0.02	2
Maximum cell concentration [cells/L]	*1–2 × 10* ^*13*^	0.5–1 × 10^11^
Maximum cell wet weight (WCW) [g/L]	200–400	*100–200*
Maximum specific growth rate [per h]	0.15	0.03

Values in italics are estimated (calculated) since they are usually not quantified for the system. The specific gravity of cells is simplified with 1

^a^Cell mass is based on the assumption of spherical cells with a diameter of 15–16 μm for CHO cells and 3–4 μm for *P. pastoris* cells

**Fig. 1:**
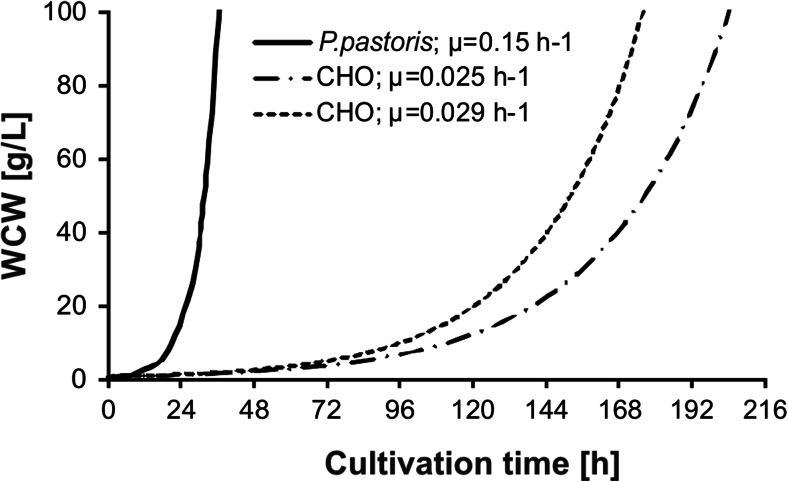
Calculated exponential growth of *P. pastoris* and two CHO cell lines. For *P. pastoris*, a starting WCW of 0.4 g/L (20 × 10^6^ cells/mL) and a *μ* of 0.15/h were assumed (*full line*), CHO cell lines were inoculated with 0.3 × 10^6^ cells/mL (0.6 g/L) and a *μ* of 0.7/day (0.029/h) (*dashed line*), and 0.6/day (0.025/h) (*chain line*) was estimated

The main and presumably the only advantage of *P. pastoris* is its high specific growth rate. Considering this advantage, Fig. 1 compares the accumulation of biomass using the *P. pastoris* system in comparison with two theoretical CHO growth curves, characterized by two different specific growth rates (0.6 and 0.7/day); *P. pastoris* was calculated with *μ* = 0.15/h. The wet cell weight (WCW) on the y-axis is limited with 100 g/L indicating the estimated exponential growth phase of the cultures.

This substantial difference in specific growth rate highlights the challenge of animal cell culture. This becomes even more critical in a large-scale 20-kL bioreactor, which needs to be filled to a maximum cell concentration of 5 × 10^10^ cells/L, meaning 10^15^ cells have to be generated from a master cell bank, which is normally frozen with 10^6^ cells/vial. This requires a process time of 29 days, and 2 tons of cells needs to be generated.

A detailed study was conducted by Maccani et al. (2014), who investigated recombinant clones of *P. pastoris* (host strain SMD1168H) and CHO (host strain DUKX-B11) expressing two different model proteins. When a scFvFc homodimeric mAb fragment (3D6scFvFc) was expressed, the maximum specific growth rate was 5–10 times higher in *P. pastoris*. The mean specific secretion rate (qP expressed as mg/g DCW/h) of 3D6scFvFc was more than 1000-fold higher in the CHO cells compared to the *P. pastoris* strains. Due to the low qP of the 3D6scFvFc in *P. pastoris* strains, the space time yield (STY; mg/L/h) was almost tenfolds lower in *P. pastoris* compared to the corresponding CHO cell line even though the maximal cell dry mass concentration (g/L) of the *P. pastoris* strain was more than 100-folds higher compared to CHO strains.

Similar conclusions were made during a direct comparison of CHO cells (DUKX-B11) with *P. pastoris* (wild-type strain X33) which recombinantly expressed a heterodimeric Fab fragment (Kunert et al. 2008). Similar to the 3D6scFvFc used by Maccani et al. (2014), simple protein-free media based on a DMEM:Ham’s F12 mixture supplemented with soy peptone and other small-molecule protein-free supplements were used for cell culture experiments.

### Current and advanced technology of mAb production

Cell culture technology has matured considerably in the last decades and evolved into a relatively reliable and robust technology. There are a number of steps to be optimized that synergistically contribute to the successful exploitation of this technology and produce a given recombinant protein at high quantity and desired quality, while keeping the production costs low.

**Fig. 2:**
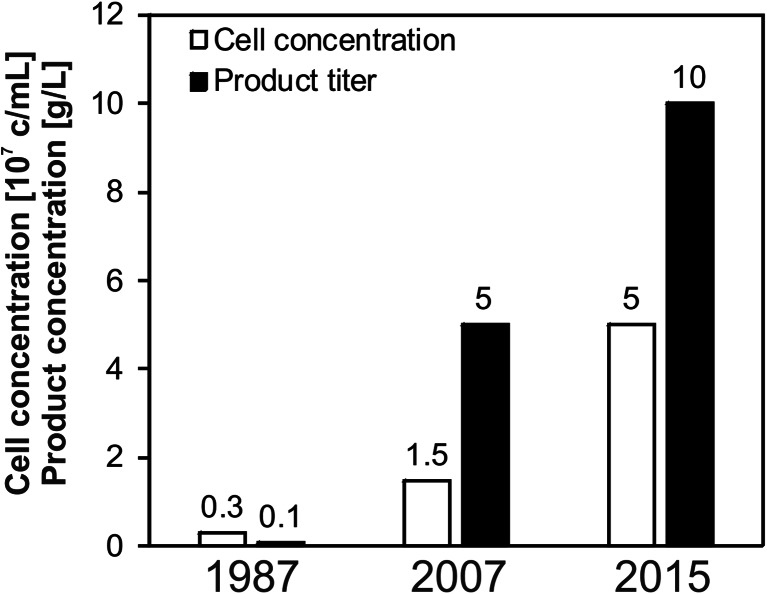
Typical cell and product concentrations achieved due to process improvements in the past three decades

One particular milestone was the development and commercial availability of improved chemically defined media that led to promising results even with rather old cell lines, which were established long before the development of the “new” media. However, higher costs and individual demands of each recombinant clone trigger the biopharmaceutical companies to develop their own media to optimize their mammalian cell technology platforms. Jayapal et al. (2007) analyzed the development of cell culture technologies and stated that almost 30 years ago, “old” bioprocesses were usually operated for a week in batch mode with peak cell concentrations of 3 × 10^6^ cells/mL and recombinant protein yields of approximately 100 mg/L (Fig. 2). Two decades later, state-of-the-art processes reached considerably longer process durations, higher cell densities, and mAb titers of 1–5 g/L due to improved basal media as well as feed strategies to replenish depleted nutrients. Nowadays, further developments such as specific feed concentrates that meet the different demands during different cultivation phases (e.g., growth and stationary phase) that enable even higher cell concentrations and product titers beyond 10 g/L are becoming reality. Recently, continuous process control strategies such as perfusion systems have started to gain popularity due to a paradigm shift of cell culture process development from pursuing even higher titers to controlling product quality and process consistency (Kelley 2009). During perfusion, a constant supply of fresh nutrients and removal of by-products can be achieved while retaining the cells in the bioreactor. This creates a rather stable environment for the product and the cells along with a short retention time of the product to preserve its quality. Additionally, such processes enable cell concentrations beyond 2 × 10^8^ cells/mL (Clincke et al. 2013) and high productivities with mAb titers up to 27 g/L (Kuczewski et al. 2011) due to product retention and concentration in the bioreactor. Compared to batch or fed-batch processes, perfusion cultures can reach several-fold higher space time yields (Table 1).

Another milestone in medium development was the move towards serum-free media. Although serum promotes cell growth, its non-standardized and complex composition is a significant source of medium variability which lowers the outcome and robustness of a bioprocess. As an alternative to serum, animal-component free protein hydrolysates derived from soy, wheat, and yeast can be added to support cell growth and productivity. However, the composition of hydrolysates also varies from lot to lot. Thus, to maximize process robustness, the implementation of fully chemically defined media is usually preferred.

Advances in monitoring and control strategies have generally improved process performance and robustness. Linking the bioprocess to the cellular metabolism can be used to implement cultivation strategies and fine-tune or direct, e.g., between cell growth and/or productivity as well as nutrient consumption and by-product accumulation (Fan et al. 2014; Reinhart et al. 2015). In general, by-product formation can be reduced at low osmolality levels and therefore increase culture viability, longevity, and recombinant protein production. However, higher osmolalities have also been shown to increase productivities (Zhang et al. 2010). Further strategies to reduce unwanted by-products such as lactic acid (mainly acidifies culture) or ammonium (toxic and negatively affects protein glycosylation) are to keep glucose or glutamine concentrations low or directly substitute them with slowly metabolized nutrients such as galactose or glutamate (Altamirano et al. 2001, 2004). Another rather simple strategy to reduce by-product formation can be realized by reduction of the cell metabolism by lowering temperature and/or pH (Trummer et al. 2006a, b).

Optimization of culture operating parameters is vitally important to obtain high product yields while meeting the quality specifications. Physical (e.g., temperature, gas flow, and impeller speed), chemical (e.g., pH, osmolality, dissolved O_2_ and CO_2_, redox potential, and metabolite levels), and biological (e.g., cell concentration, viability, cell cycle, mitochondrial activity, and NADH and LDH levels) parameters can all significantly affect product quality and potency, especially with respect to glycosylation, post-transcriptional modifications, and impurity profiles (Li et al. 2010b). These factors can cause substantial variability during bioprocessing. Little understanding of the sources of variation in mammalian cell culture processes is certainly one of the challenges ahead. Currently, a better understanding of bioprocesses towards ensuring final product quality is driven by the US Food and Drug Administration’s (FDA) process analytical technology (PAT) initiative and possibly links process parameters and reproducibility, culture performance, and product quality (FDA 2004).

Disposable technologies are continuously gaining more importance and are nowadays no longer limited to laboratory scale but are continuously being applied on a larger scale. Compared to traditional stainless steel tanks, single-use bioreactors offer numerous advantages such as lower initial investment costs, an increased reliability, and no validation efforts as they arrive clean and pre-sterilized; minimal requirements for installation, qualification, and personnel training and can increase plant capacity and flexibility by reducing turnaround and set-up time, with reduced piping, valve, and instrumentation requirements as well as faster implementation of design changes allowing for continuous improvement and integration of new technologies such as on-line monitoring systems (Li et al. 2010b). Improved on-line and off-line sensors are capable of providing information that further enhances process monitoring and control. The first generation of on-line probes for optical cell density quantification often showed erroneous readings at decreasing viabilities at later stages of bioprocesses. Next-generation probes rely on dielectric permittivity and electrical impedance spectroscopy and are more accurate to determine viable cell volume (Zeiser et al. 1999; Downey et al. 2014). Spectroscopic probes, based on near- or mid-infrared range, allow non-invasive assessment of complex cell culture media components such as nutrients, by-products, viability, and cell and recombinant product concentrations (Li et al. 2010a) directly in the bioreactor allow even better process control in real time.

## The factor “product quality” in mAb production

The primary sequence of a mAb expressed in different cell systems is mainly determined by the codons of the transgene, but post-translational modifications determine a recombinant protein’s microheterogeneity and have a significant impact on its solubility, stability, pharmacokinetics, potency, and biological activities. Enzymatic modifications start with the cleavage of the signal sequence and follow the path of protein maturation in the ER and the Golgi. The aim of therapeutic protein manufacturing is to generate proteins’ microheterogeneity as close to the in vivo situation as possible. But especially for polyclonal immunoglobulins from human serum, this is a very complex picture despite the Fc fragment of all four IgG subclasses carrying only a single highly conserved N-glycosylation site. The predominant structure of these oligosaccharides is a biantennary complex-type structure, mostly core-fucosylated partially with a bisecting N-acetylglucosamine, and a small portion may carry one or two sialic acid residues on the antennae (Raju 2008). The most significant variation is determined by the degree of galactosylation and a varying portion of truncated glycoforms. Various studies have indicated that the glycosylation profile in particular changes during age or specific physiological and pathological conditions (Parekh et al. 1985; Yamada et al. 1997; Chen et al. 2012). Therefore, it is necessary to focus on commonly described and discussed specific differences of glycosylation like fucosylation, sialylation, occurrence of bisecting N-acetylglucosamine, and the degree of galactosylation.

*Galactosylation* is rather important for mediating complement-dependent cytotoxicity (CDC), and different studies confirmed the positive effect of fully galactosylated IgG due to increased antibody binding to C1q (Jefferis 2009). In contrast, IgG half-life is not supported by terminal galactose residues (Jones et al. 2007).

*Alpha1*,*6-fucosylation* is often discussed because of its role in antibody-dependent cell-mediated cytotoxicity (ADCC) initiated by binding of the specific antibody to the FcγRIIIa receptor. Despite the fact that more than 80 % of serum IgG is fucosylated, it has been found that non-fucosylated IgG’s increase ADCC significantly (Miyoshi et al. 1999; Xue et al. 2013). Since CHO cell-expressed mAbs contain 90 % fucosylated carbohydrates, host cells are genetically engineered to lack α1,6-fucosyltransferase (Satoh et al. 2006).

The *bisecting N-acetyl-glucosamine*, bound to the central mannose residue, is another important sugar moiety that is known to improve ADCC. CHO and NS0 cells do not express N-acetyl-glucosaminyl transferase III (Umaña et al. 1999), and therefore, different groups aim to generate appropriate cell lines (Ferrara et al. 2006).

*Sialylation* might have a beneficial effect on serum half-life (Raju and Lang 2014), but it has also been reported that terminal sialic acid reduces ADCC (Scallon et al. 2007).

Beneath the highly inhomogeneous picture of glycosylation of mAbs and serum IgG, chemical or enzymatic modifications of amino acids have been described. N-terminal glutamic acid and glutamine might be converted to pyroglutamate and C-terminal lysine can be cleaved off (Brorson and Jia 2014). Cyclic pyroglutamate not only was found in vivo but also occurs biologically after long bioprocess duration and chemically during purification, formulation, storage, or even under analytical procedures. After lysine cleavage, the newly generated terminal proline residue is now susceptible to another modification, namely amidation. Plasma-derived antibodies generally lack C-terminal lysine residues in their heavy chains (Harris 1995). Terminal modifications were not estimated as having an impact on antigen or receptor binding, but from a regulatory perspective, they should be considered in risk-assessments and the impact on product quality and safety should be studied.

Deamidation of asparagine and glutamine is sometimes found in the antigen binding site of mAbs and was evaluated to have a major impact on the binding affinity (Huang et al. 2005; Haberger et al. 2014). Other modifications like oxidation of methionine (Liu et al. 2008), the formation of disulfide bonds, or glycation (Quan et al. 2008) may also lead to microheterogeneity being defined as a critical quality attribute and should be controlled carefully (Goetze et al. 2010).

The quality of mAbs needs to be controlled and assured for the final product formulation until to the moment of in vivo application. Biophysical properties, the heterogeneity in size, and the net charge of the molecule are important parameters. In-depth analysis of glycosylation variants are performed by mass spectroscopy (MS). Due to the expensive equipment and the demand of highly specialized scientists, it is assumed that in the near future, MS will be performed by designated protein biochemists. However, since DoE protocols call for multitudes of analyzed samples alternative techniques are adapted to trace enzymatic and chemical changes to indicate the mAb microheterogeneity. Such methods are often based on protein size, surface charge, or the combination of both properties.

Protein aggregation and formation of particles commonly occur during the bioprocess by structural, environmental, and processing factors. The therapeutic protein can thereby lose biological activity, develop immunologic reactions, or other side effects. Therefore, analyzing product quality early in development is necessary to rapidly match the desired quality profile. A broad range of size (nm–μm) has to be considered and complimentary, orthogonal analytical techniques are necessary to verify the results based on different principles and setups (Telikepalli et al. 2014). Size exclusion chromatography (SEC) is a powerful tool to analyze smaller aggregates in the size range of tens of nanometers but small nanometer-sized soluble aggregates might dissociate by interacting with the mobile phase or adhere to the column. Therefore, HIC-HPLC was developed and gave promising results for the analysis of different mAb isoforms including aggregates (Haverick et al. 2014). Alternatively, SEC was applied in an HPLC format to improve isoform resolution (Paul et al. 2014). Nanoparticle tracking analysis (NTA) is able to analyze submicron particles (0.1 to 1 μm) by combining laser light scattering microscopy with a charge-coupled device camera enabling the visualization and recording of nanoparticles in solution. The complementary method is dynamic light scattering (DLS) which determines particle size from fluctuations in scattered light intensity due to the Brownian movement of the particles, but accurate size determination is rather critical with this method. Electron microscopy analysis provides highly detailed information not only about particle size but also about particle shape and surface texture. Currently, a common application is limited since equipment is expensive and analyses rely on microscope experts. The size distribution determined by analytical ultracentrifugation (AUC) is also more complex and needs to be combined with the above-mentioned methods, such as PAGE and SEC including all their uncertainties.

Analysis of charge variant distribution of mAb preparations gives insight to chemical and enzymatic modifications of mAbs that may result from the bioprocess or the formulation/storage conditions (Lingg et al. 2014). Conventional ion-exchange chromatography needs to be elaborated for every single mAb, is a time-consuming procedure, and needs several micrograms of mAb for each injection. This led to the development of highly sensitive alternatives like capillary ion-exchange chromatography (Rea et al. 2015) and high-performance ion-exchange chromatography (Kishishita et al. 2015) to analyze acidic, main, and basic mAb fractions. To handle multiple samples in a short time and evaluate different process strategies, Wheeler et al. (2014) developed a microchip zone electrophoresis for high throughput analysis of mAbs.

## Conclusion

**Fig. 3:**
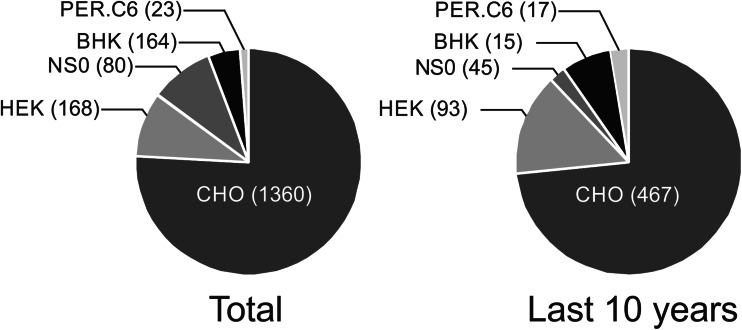
Score of scientific publications in PubMed (US National Library of Medicine) using diverse popular cell lines for recombinant protein expression in the last 10 years as well as total results

By summarizing biological and technological aspects for recombinant mAb production in different mammalian cell culture hosts, it becomes clear that despite their long scientific history a lot of development work is still invested in them. The number of scientific publications containing “CHO” in the title and text in PubMed (US National Library of Medicine) far exceeds other commonly used cell lines for recombinant protein expression (Fig. 3). Approximately 75 % of scientific publications have used CHO cells since the beginning, and this percentage has remained unchanged for the last 10 years. Therefore, CHO cells will probably remain the workhorse for protein expression in mammalian cell culture in the next decades too. In parallel, much work was and will be put into the development of alternative expression systems especially for mAb variants without in vivo counterparts, new conjugated formats, or artificial molecules. These may even be expressed in lower eukaryotes or prokaryotes, depending on if they can meet the desired quality specifications. Another point to consider is the development of biosimilars where the community has experienced difficulties in meeting the branded original without precisely mimicking the whole production of the original drug.
